# Force decay of polyethylene terephthalate glycol aligner materials during simulation of typical clinical loading/unloading scenarios

**DOI:** 10.1007/s00056-021-00364-5

**Published:** 2021-12-09

**Authors:** Fayez Elkholy, Silva Schmidt, Falko Schmidt, Masoud Amirkhani, Bernd G. Lapatki

**Affiliations:** 1 Department of Orthodontics, University of Ulm, Albert-Einstein-Allee 11, 89081 Ulm, Germany; 2 Institute of Experimental Physics, University of Ulm, Albert-Einstein-Allee 11, 89081 Ulm, Germany

**Keywords:** Orthodontic appliances, Aligner, Force, Stiffness, Thermoplastic, Orthodontic therapy, Kieferorthopädische Apparaturen, Aligner, Kraft, Steifigkeit, Thermoplastische, Kieferorthopädische Therapie

## Abstract

**Background:**

This in vitro study investigated the effect of three distinct daily loading/unloading cycles on force delivery during orthodontic aligner therapy. The cycles were applied for 7 days and were designed to reflect typical clinical aligner application scenarios.

**Materials and methods:**

Flat polyethylene terephthalate glycol (PET-G) specimens (Duran®, Scheu Dental, Iserlohn, Germany) with thicknesses ranging between 0.4 and 0.75 mm were tested in a three-point-bending testing machine. Measurements comprised loading/unloading intervals of 12 h/12 h, 18 h/6 h, and 23 h/1 h, and specimens were exposed to bidistilled water during loading to simulate intraoral conditions.

**Results:**

A very large decay in force for the PET‑G specimens could already be observed after the first loading period, with significantly different residual force values of 24, 20, and 21% recorded for the 12 h/12 h, 18 h/6 h, and 23 h/1 h loading/unloading modes, respectively (Mann–Whitney *U* test, *p* < 0.01). In addition, further decays in force from the first to the last loading period at day 7 of 13.5% (12 h/12 h), 9.7% (18 h/6 h), and 8.4% (23 h/1 h) differed significantly among the three distinct loading modes (Mann–Whitney *U* test, *p* < 0.01).

**Conclusion:**

Although the initial material stiffness of PET‑G is relatively high, the transmission of excessive forces is attenuated by the high material-related force decay already within a few hours after intraoral insertion.

## Abstract (de)

**Hintergrund:**

In dieser In-vitro-Studie wurden die Auswirkungen von 3 unterschiedlichen täglichen Be- und Entlastungszyklen auf die Kraftübertragung während der kieferorthopädischen Zahnbewegung untersucht. Die Zyklen wurden 7 Tage lang angewendet und waren auf typische klinische Aligner-Anwendungsszenarien hin konzipiert.

**Material und Methoden:**

Plane PET-G(Polyethylenterephthalatglykol)-Proben (Duran®, Scheu Dental, Iserlohn, Deutschland) mit einer Dicke zwischen 0,4 und 0,75 mm wurden in einer Dreipunktbiegeprüfmaschine getestet. Die Messungen umfassten Belastungs‑/Entlastungsintervalle von 12 h/12 h, 18 h/6 h und 23 h/1 h. Zur Simulation der intraoralen Bedingungen wurden die Probekörper während der Belastung bidestilliertem Wasser ausgesetzt.

**Ergebnisse:**

Bei den PET-G-Proben war bereits nach der ersten Belastungsperiode eine sehr starke Kraftabnahme zu beobachten, wobei für die Be‑/Entlastungsmodi 12 h/12 h, 18 h/6 h bzw. 23 h/1 h signifikant unterschiedliche Restkraftwerte ermittelt wurden: 24, 20 bzw. 21% (Mann-Whitney-U-Test, *p* < 0,01). Darüber hinaus unterschieden sich die weiteren Kraftabfälle von der ersten bis zur letzten Belastungsperiode am Tag 7 mit 13,5% (12 h/12 h), 9,7% (18 h/6 h) und 8,4% (23 h/1 h) signifikant zwischen den 3 unterschiedlichen Belastungsmodi (Mann-Whitney-U-Test, *p* < 0,01).

**Schlussfolgerung:**

Obwohl die initiale Materialsteifigkeit von PET‑G relativ hoch ist, wird die Übertragung überhöhter Kräfte durch den hohen materialbedingten Kraftabbau innerhalb weniger Stunden nach dem intraoralen Einsetzen abgeschwächt.

## Background

Interest in aligners as an alternative treatment for correction of tooth malpositions and malocclusions continues to grow. The main concept of aligner therapy is based on incremental correction of a tooth’s malposition, with each increment corresponding to a single setup model on which an aligner or a set of aligners is fabricated. Aligners generate tooth movement that results from the discrepancy between the actual tooth position and the programmed tooth position on the setup model. This discrepancy causes local deformation of the aligner and generates contact forces between the aligner and teeth [4–7, 9, 13, 15–17]. Since the introduction of the principle of aligner therapy by Kesling, different materials and concepts have been developed [19]. With regard to the setup increment sizes and material thicknesses used, two fundamentally different approaches are applied. The first approach is based on very small setup increments (range 0.1–0.2 mm) and uses identical aligners for the same movement increment; e.g., Invisalign® system (Align Technology, Santa Clara, CA, USA). The second approach is based on larger movement increments (range 0.5–1.0 mm) and uses a sequence of aligners of ascending thickness within each increment.

The forces and moments applied to individual teeth by aligners are affected by several material-dependent variables such as the initial stiffness of the aligner and the behavior of the aligner material during its period of intraoral application (usually 1–2 weeks) [8, 20]. Several experimental studies have used multi-axial sensors to investigate the force–moment systems delivered by aligners on central incisors or canines depending on the kind of movement (i.e., tipping, derotation, and bodily movement) [4–7, 15–17]. These studies generally indicated that the recommended combinations of setup increments and material thicknesses produce force and moment magnitudes significantly in excess of those considered sufficient for orthodontic tooth movement [22]. Such overloading of the periodontal ligament (PDL) is known to increase the risk of orthodontically induced irreversible root resorption [22]. It is important to note, however, that the force–moment systems generated by aligners during clinical application are subject to a certain deterioration related to the physiological mobility of the tooth within the PDL, orthodontic tooth movement, and the stress relaxation properties of aligner materials [4, 20].

Several studies have investigated this third aspect by using three-point-bending tests and flat polyethylene terephthalate glycol (PET-G) or thermoplastic polyurethane (TPU) specimens to evaluate the effect of different durations of constant loading on the mechanical properties of aligners [10, 18, 21]. In two of these studies, specimens were only loaded for 30–180 min, which is unrepresentative of the typical long use periods for aligners of up to 2 weeks [10, 18]. Nevertheless, the results showed that the initially determined stresses decreased by approximately 22.9% after 30 min of constant loading, and by 44.5% after 180 min [10]. Other studies were performed for longer constant loading periods of up to 24 h, and residual forces of 56 and 38% were observed for PET‑G specimens after 8 h and 24 h of constant loading, respectively [21]. Similar patterns were reported for the TPU specimens with residual forces of 59.5 and 45.5% for the 8‑ and 24-hour loading periods, respectively [21]. Based on these results, it was concluded that the forces applied by PET‑G aligners reduce substantially during the first 8 h of loading and then reach a plateau. It must be noted, however, that the constant loading pattern applied in these previous studies did not account for the fact that aligners are not worn by the patient constantly, but are usually inserted and removed several times throughout the day [25]. The specific intraoral application schedule of a patient, in turn, might affect the force delivery of the aligner material and, therefore, the force–moment systems applied to individual teeth during aligner therapy. The aim of this study was to evaluate the effect of different loading/unloading intervals on the mechanical properties of thermoformed PET‑G aligner materials in a simulated intraoral environment during a 1-week observation period. We hypothesized that longer loading cycles would result in greater stress relaxation and larger reductions in force than shorter loading cycles.

## Materials and methods

### Test apparatus

**Fig. 1:**
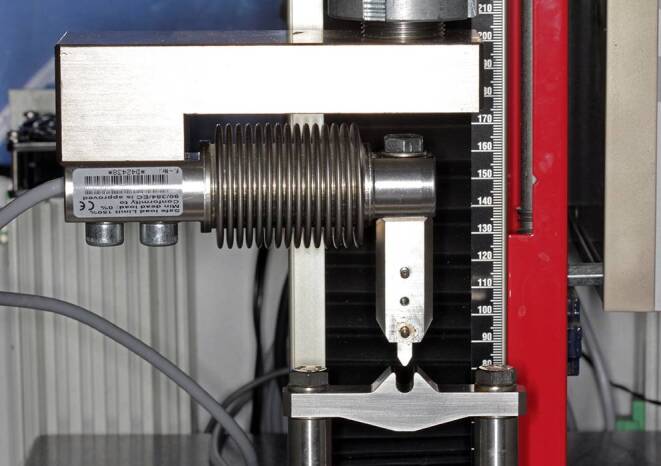
Detailed view of the three-point-bending test setup with 8 mm span length and one-dimensional force sensor used Detailansicht des Dreipunktbiegeversuchsaufbaus mit 8‑mm-Spannweite und eindimensionalem Kraftsensor

**Fig. 2:**
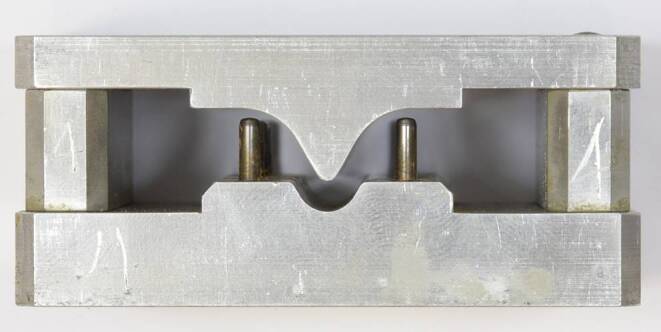
Specially designed three-point-bending, stainless-steel device for bending specimens during the corresponding long loading periods. Each device accommodated three specimens Speziell entworfene Dreipunktbiegevorrichtung aus Edelstahl zum Biegen der Proben während der entsprechenden langen Belastungszeiten. Jede Vorrichtung enthielt 3 Probekörper

Measurements were performed in a three-point-bending setup integrated in a universal material testing machine equipped with a 100 N load sensor (Z2.5, Zwick-Roell, Ulm, Germany). The lateral supports and central stylus had a radius of 0.5 mm with a total span length of 8 mm (Fig. 1). The whole setup was enclosed in a climate chamber to maintain a temperature of 37 °C during measurements. This testing machine was only occupied during the bending force measurements. For long-term loading, specimens were mounted in 12 specially designed “loading devices”, i.e., three-point-bending, stainless-steel devices accommodating three specimens each (Fig. 2). They consisted of two shells bilaterally separated by identical spacer blocks. Their lateral supports and stylus were identical in geometry to those of the testing machine. To accurately adjust the predefined, thickness-dependent bending depths and compensate for the thickness reduction of the specimens after thermoforming, precision feeler gauges (T3525M, Carl Kammerling International Ltd., Glanydon, United Kingdom) with specific thicknesses were selected and positioned bilaterally on the spacer blocks before the two shells were assembled by means of fixation screws (Fig. 2).

### Aligner specimens and preparation

**Fig. 3:**
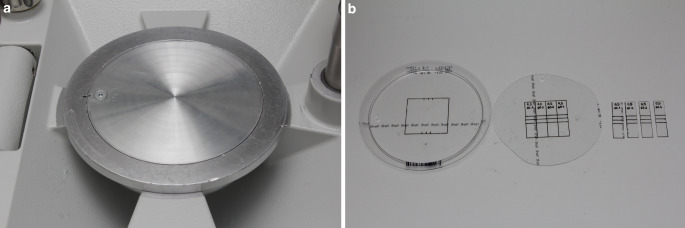
**a** Flat metal plate used to thermoform the polyethylene terephthalate glycol (PET-G) films. **b** Steps of specimen preparation. A cutting template was used to mark the cutting lines and middle of each specimen **a** Flache Metallplatte, verwendet zum Thermoformen / Tiefziehen der PET-G(Polyethylenterephthalatglykol)-Folien. **b** Schritte der Probenvorbereitung. Eine Schnittschablone wurde verwendet, um die Schnittlinien und die Mitte jedes Probekörpers zu markieren

Test specimens were fabricated from PET‑G films (Duran®, Scheu Dental GmbH, Iserlohn, Germany) with nominal thicknesses of 0.4, 0.5, 0.625, and 0.75 mm. All films were thermoformed on a specially fabricated flat metal plate to simulate, in a reproducible setting, the laboratory process used for aligner production (Fig. 3a). The selection of the metal plate was based on the method described in a previous study indicating similar force reduction for both aligner films thermoformed on a flat metal plate and on a stone cylinder form when compared to the specimens extracted from raw untreated aligner films [8].

All films were thermoformed according to the manufacturer’s recommendations. Four rectangular specimens measuring 10 × 40 mm were then extracted from the central region of each thermoformed film (Fig. 3b). Before specimen extraction, two more lines defining the exact position of the lateral supports were marked at a distance of ±4 mm from and parallel to the midline. This ensured reproducible positioning of the specimens in the measurement apparatus and loading devices. The thickness of each specimen was determined after thermoforming by means of a digital micrometer screw gauge (Toolcraft, Georgensgmünd, Germany).

### Test procedure

**Fig. 4:**
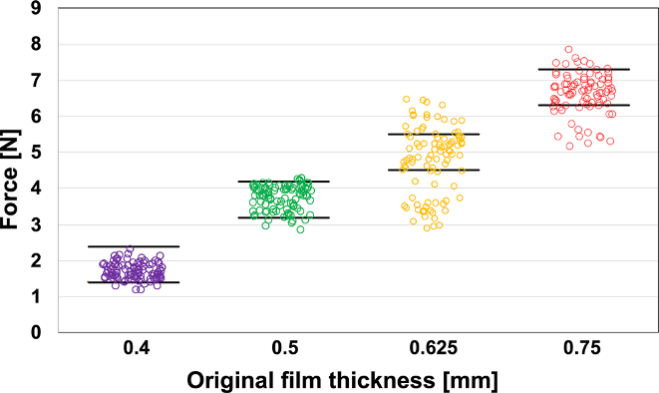
Initial forces measured for multiple specimens of different thicknesses to determine variation within the same specimen thickness. Only specimens with bending forces of ±0.5 N around the median value (*black horizontal lines*) were included in further tests Die Ausgangskräfte wurden für mehrere Proben unterschiedlicher Dicke gemessen, um Schwankungen innerhalb der gleichen Probendicke zu ermitteln. Nur Proben mit Biegekräften von ±0,5 N um den Medianwert (*schwarze horizontale Linien*) wurden in die weiteren Prüfungen einbezogen

Before long-term loading, the initial reference load for each specimen was measured under dry conditions during thickness-dependent deflection in the testing machine. During these measurements, we noticed a certain inter-specimen variability of the bending forces recorded for different specimens of the same thickness. This was, however, an expected variability due to the material production as well as the thermoforming process [8]. To control this variability and exclude outliers and their influence on the test results, only specimens within ±0.5 N of the median value for a specific thickness were included in the further tests (Fig. 4). After determining their initial bending forces at the predetermined deflection ranges, specimens were loaded in the described loading devices and stored in a bath filled with bidistilled water heated to 37 °C.

To simulate different daily schedules of aligner use during clinical therapy, three experimental loading and unloading modes consisting of seven daily cycles each were tested: (a) 23-hour loading and 1‑hour unloading (23 h/1 h), (b) 18-hour loading and 6‑hour unloading (18 h/6 h), and (c) 12-hour loading and 12-hour unloading (12 h/12 h). After each loading period, specimens were removed from the device and a short force measurement (F_L_) was performed in the testing machine immediately after reaching the maximum deflection. Specimens were then stored in a dry environment at room temperature for the designated unloading period before the second force measurement (F_U_) was performed for the corresponding loading/unloading cycle. Subsequently, the specimen was again mounted in the loading device and the entire assembly immersed in the water bath to enter the following daily cycle.

Three specimens were tested for each of the four film thicknesses and three loading/unloading modes, resulting in 36 tested specimens in total.

### Data analysis

**Fig. 5:**
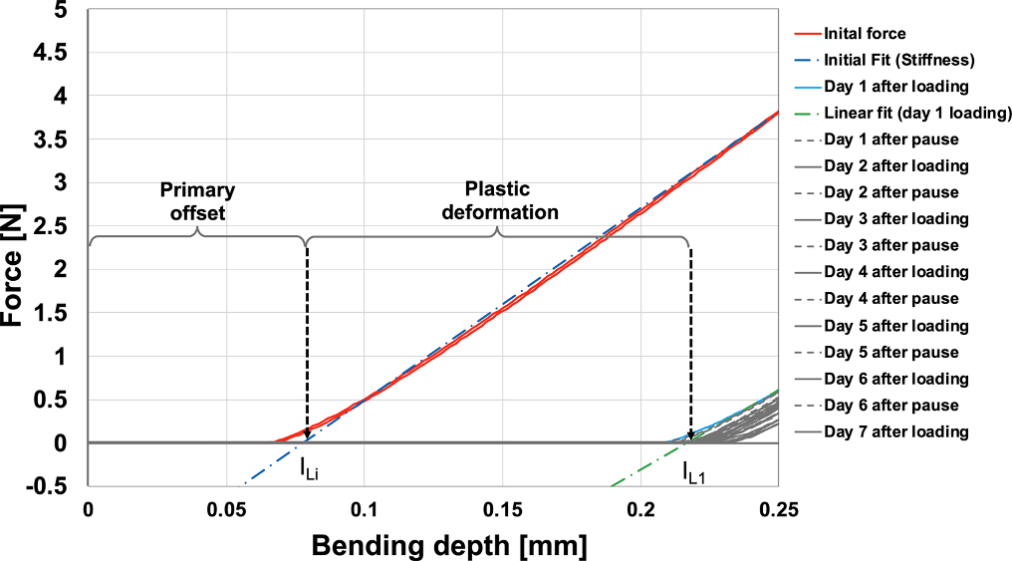
Measurement curves obtained for one Duran® 0.5 mm specimen during the 7‑day study period of seven loading and seven unloading cycles. The *red curve* represents the force/deflection curve for the initial measurement and the *grey curves* represent the consequent loading/unloading measurements over the 7‑day observation period. The *dashed lines* represent the linear fits for determining the stiffness of the specimen at different time points. The primary offset describes the distance between the initial position of the stylus and its first contact with the specimen. The further shift of the curves represents the plastic deformation of the specimens Messkurven, die für eine 0,5-mm-Duran®-Probe während des 7‑tägigen Untersuchungszeitraums mit 7 Belastungs- und 7 Entlastungszyklen ermittelt wurden. Die *rote Kurve* stellt die Kraft‑/Durchbiegungskurve für die erste Messung dar, die *grauen Kurven* die aufeinander folgenden Be‑/Entlastungsmessungen während des 7‑tägigen Beobachtungszeitraums. Die *gestrichelten Linien* stellen die linearen Anpassungen zur Bestimmung der Steifigkeit des Probekörpers zu verschiedenen Zeitpunkten dar. Die primäre Verschiebung beschreibt den Abstand zwischen der Anfangsposition des Tastereinsatzes und seinem ersten Kontakt mit der Probe, die weitere Verschiebung der Kurven stellt die plastische Verformung der Proben dar

The registered force–deflection curves were evaluated by using algorithms programmed in Matlab® (MathWorks, Natick, MA, USA). Fig. 5 shows the force–deflection behavior of a 0.5-mm PET‑G specimen during seven 23 h/1 h loading/unloading cycles. The unloaded specimen showed an initial offset that was quantitatively determined as the intersection between the corresponding linear fit of the initial force curve and the x‑axis (I_Li_). The slope of this fit indicates the initial stiffness of the specimen. A linear fit was also applied to determine stiffness values after each of the subsequent six loading and unloading cycles. The distance between (i) their intersection with the x‑axis and (ii) point (I_Li_) describes the specimen’s plastic deformation for the corresponding daily cycle.

Statistical analysis was performed using the software R (R Foundation for Statistical Computing, Vienna, Austria).

Data were pooled in two different manners. To determine any thickness-dependent or loading-mode-dependent changes, a Mann–Whitney *U* test was performed. For mode-dependent comparisons, we pooled the different specimen thicknesses according to the loading/unloading mode used. Here, only the percentage force or plastic deformation change was compared. In this manner, we were able to overcome the influence of the inter-specimen variability within the same thickness and to increase the reproducibility of the recorded results. Accordingly, we pooled the data for different loading/unloading modes for each specimen thickness to determine any thickness-dependent effect. Furthermore, the power analysis of the Mann–Whitney *U* test for the observed thickness-dependent or loading-mode-dependent effects on the measured forces was performed using Monte Carlo simulation generating 10,000 sets of data (Table 1). The sample size calculation was also simulated accordingly for different sample numbers until a power of >0.9 was achieved.

**Table 1:** Results of the power analysis of the Mann–Whitney *U* test for the observed thickness-dependent or loading-mode-dependent effects for the relation of the forces after the first (L1), fourth (L4) and seventh (L7) loading cycle related to the initially measured forces (L0) Ergebnisse der Power-Analyse des Mann-Whitney-U-Tests für die beobachteten dicken- bzw. belastungsartabhängigen Effekte für das Verhältnis der Kräfte nach dem ersten (L1), vierten (L4) und siebten (L7) Belastungszyklus bezogen auf die ursprünglich gemessenen Kräfte (L0)

Compared parameter	Sample size	Statistical power		
		L0/L1	L0/L4	L0/L7
Loading-mode-dependent force changes	*n* = 3	0.913	*0.004*	*0.001*
Thickness-dependent force changes		1.000	1.000	1.000
Loading-mode-dependent force changes	*n* = 17	1.000	0.928	0.9793
Thickness-dependent force changes		1.000	1.000	1.000

## Results

### Power analysis and sample size calculation

According to the sample size calculation, the tested sample size (*n* = 3) was sufficient for demonstrating the force reduction after the first loading period for all thicknesses and loading modes with a power value >0.9. Due to the nearly similar force reduction values and relatively high variability a minimum of 17 samples would have been required to show a statistically significant (power >0.9) difference between the different modes along the further loading cycles.

### Force decay after loading periods

**Fig. 6:**
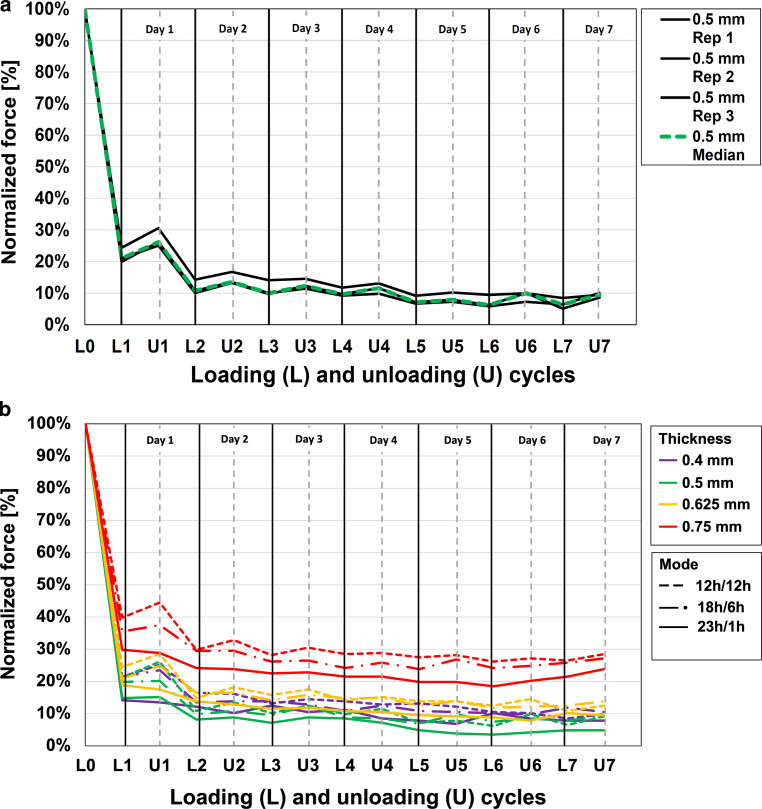
**a** Residual forces obtained for the three 0.5-mm specimens investigated during the 12 h/12 h loading/unloading mode. The *green dashed line* represents the median curve and the *solid lines* represent the values for the individual specimens. **b** Corresponding median residual forces for other film thicknesses and different loading/unloading modes **a** Residualkräfte für die drei 0,5-mm-Proben, die während des Belastungs‑/Entlastungsmodus 12 h/12 h untersucht wurden. Die *grün gestrichelte Linie *stellt die mittlere Kurve dar, die *durchgezogenen Linien* die Werte für die einzelnen Proben. **b** Entsprechende mittlere Residualkräfte für andere Schichtdicken und unterschiedliche Be‑/Entlastungsmodi

Fig. 6a shows the individual curves obtained for three 0.5-mm specimens exposed to the 12 h/12 h loading/unloading mode. Force values decreased greatly after the first loading cycle (L1) to a median residual value of 21.1% (range 20.6–24.0%). Progressively smaller reductions were observed after subsequent loading intervals until a near-constant median residual force level of 9.7% was reached. Although the 12 h/12 h mode also resulted in a clear force reduction in other specimen thicknesses, this effect was less pronounced for the 0.75-mm specimen (Fig. 6b), reaching only 39.8, 28.6, and 28.5% after the first (L1), fourth (L4), and last (L7) loading intervals, respectively (Table 2). Forces for the other two loading/unloading modes (18 h/6 h and 23 h/1 h) were quite similar after the six loading periods. However, the median force curves for all three modes and four specimen thicknesses (Fig. 6b) illustrate that the reduction of initial residual force increased significantly as the length of the loading interval also increased (Mann–Whitney *U* test, *p* < 0.01). This trend is quantitatively proved by the distinct median differences for L1–L7 of 13.5, 9.7 and 8.4% for the 12 h/12 h, 18 h/6 h, and 23 h/1 h modes, respectively.

**Table 2:** Initial and normalized residual forces for the different specimen thicknesses. The values 12 h/12 h, 18 h/6 h, and 23 h/1 h denote the different loading/unloading modes, i.e., 12 h/12 h, 18 h/6 h, and 23 h/1 h, respectively. The values in parentheses correspond to the maximum and minimum values for the corresponding force in newtons and normalized residual force in percent Initiale und normierte Residualkräfte für die verschiedenen Probekörperdicken. Die Werte 12 h/12 h, 18 h/6 h und 23 h/1 h bezeichnen die verschiedenen Be‑/Entlastungsmodi, d. h. 12 h/12 h, 18 h/6 h und 23 h/1 h. Die Werte in Klammern entsprechen den Maximal- und Minimalwerten für die entsprechende Kraft in Newton und die normierte Restkraft in Prozent

Original film thickness		0.4 mm			0.5 mm			0.625 mm			0.75 mm		
*Loading/unloading mode*		12 h/12 h	18 h/6 h	23 h/1 h	12 h/12 h	18 h/6 h	23 h/1 h	12 h/12 h	18 h/6 h	23 h/1 h	12 h/12 h	18 h/6 h	23 h/1 h
*Initial force [N]*		2.03 (2.12–2.00)	1.92 (2.00–1.89)	1.63 (1.67–1.56)	4.09 (4.09–3.97)	3.91 (4.07–3.87)	3.82 (3.83–3.81)	5.38 (5.44–5.30)	5.29 (5.42–5.08)	5.33 (5.44–5.04)	6.83 (7.00–6.69)	7.15 (7.24–6.89)	6.86 (6.94–6.85)
*Normalized forces [%]*	*L0*	100%	100%	100%	100%	100%	100%	100%	100%	100%	100%	100%	100%
	*L1*	21.6% (21–24)	21.5% (15–22)	14.0% (14–16)	21.1% (20–24)	19.7% (17–20)	14.7% (14–16)	24.6% (22–26)	20.8% (20–23)	18.8% (16–20)	39.8% (32–41)	35.4% (31–38)	29.7% (25–35)
	*U1*	25.2% (24–27)	23.3% (18–24)	13.4% (12–15)	26.1% (25–31)	20.2% (20–21)	15.1% (13–16)	28.3% (26–30)	24.9% (22–26)	17.3% (15–19)	44.4% (38–46)	37.5% (33–40)	29.0% (25–35)
	*L2*	16.6% (13–18)	13.5% (8–15)	12.0% (10–14)	10.8% (10–14)	9.6% (9–11)	8.2% (8–12)	14.7% (14–16)	16.5% (14–17)	13.7% (12–15)	29.7% (23–34)	29.3% (22–32)	24.2% (21–30)
	*U2*	16.3% (15–18)	13.6% (11–15)	10.1% (10–13)	13.5% (13–17)	10.7% (9–13)	8.9% (8–13)	18.2% (18–20)	16.3% (16–17)	12.7% (12–14)	32.8% (26–37)	29.4% (24–34)	23.7% (19–28)
	*L3*	13.2% (12–15)	13.6% (11–16)	12.5% (12–13)	10.0% (10–14)	9.3% (8–10)	7.2% (6–14)	15.7% (15–17)	14.2% (14–15)	11.5% (9–14)	28.1% (20–31)	26.1% (19–29)	22.5% (18–26)
	*U3*	14.5% (13–15)	12.9% (10–14)	10.5% (10–12)	12.3% (11–15)	12.3% (9–12)	8.7% (7–12)	17.3% (16–18)	15.8% (15–16)	11.8% (10–17)	30.5% (23–33)	26.5% (20–31)	22.8% (18–26)
	*L4*	13.6% (11–15)	10.8% (8–11)	11.1% (9–13)	9.7% (9–12)	8.8% (6–9)	8.3% (6–14)	14.0% (14–14)	14.8% (12–15)	10.7% (10–13)	28.6% (21–30)	24.1% (18–28)	21.4% (18–24)
	*U4*	12.9% (11–15)	12.5% (8–14)	8.6% (8–10)	11.6% (10–13)	8.4% (8–9)	7.0% (6–12)	15.1% (15–15)	14.7% (12–15)	10.3% (9–12)	28.9% (23–32)	25.7% (21–30)	21.4% (16–22)
	*L5*	13.0% (12–13)	10.7% (8–13)	7.7% (7–10)	7.1% (7–9)	6.6% (6–10)	4.7% (4–9)	13.6% (12–14)	12.3% (9–13)	9.6% (8–13)	27.5% (20–29)	23.6% (17–27)	19.8% (15–22)
	*U5*	12.0% (11–12)	10.4% (8–15)	6.8% (7–7)	7.9% (7–10)	9.8% (7–10)	3.8% (3–11)	13.8% (12–15)	13.7% (11–15)	9.2% (9–12)	28.0% (22–32)	26.9% (19–28)	19.7% (16–22)
	*L6*	10.4% (9–12)	10.0% (6–12)	10.0% (7–10)	6.2% (6–9)	7.5% (7–9)	3.3% (3–9)	12.4% (8–14)	11.6% (11–13)	8.7% (8–12)	26.3% (18–29)	24.0% (18–28)	18.5% (15–22)
	*U6*	10.1% (10–11)	9.9% (7–11)	8.3% (7–8)	9.6% (7–10)	8.5% (7–9)	4.2% (2–6)	14.5% (9–15)	12.0% (10–14)	7.7% (7–11)	27.2% (21–31)	24.9% (20–29)	20.3% (15–20)
	*L7*	8.6% (8–10)	11.6% (9–12)	7.8% (7–8)	6.4% (5–8)	7.6% (7–8)	4.8% (4–9)	10.9% (7–13)	12.6% (11–15)	10.0% (8–11)	26.4% (21–28)	25.9% (18–29)	21.6% (16–24)
	*U7*	9.6% (7–11)	10.3% (9–11)	7.8% (7–8)	9.2% (9–10)	9.6% (7–10)	4.7% (3–7)	12.4% (9–16)	13.8% (13–18)	9.4% (8–12)	28.5% (23–29)	27.2% (20–30)	23.7% (18–26)

### Force recovery after unloading periods

Fig. 6a shows that the deflection forces for the three 0.5-mm specimens exposed to the 12 h/12 h mode increased after the unloading intervals. Nevertheless, this “force recovery” progressively decreased after each daily cycle (Table 2). Correspondingly, the median force increase was 5.1% after the first unloaded cycle (U1) and reached values of 1.9 and 2.7% after U4 and U7, respectively (Fig. 6a and Table 2). A similar pattern was observed for the other specimen thicknesses during the 12 h/12 h loading/unloading mode, with average force recovery values of 4.0, 0.6, and 1.5% after U1, U4, and U7, respectively (Fig. 6b and Table 2). Increasing the loading time from 12 to 18 h (18 h/6 h mode) did not result in a significantly different force-recovery behavior after the fourth and seventh unloading periods (U4 and U7; Mann–Whitney *U* test, *p* > 0.05). For the 23 h/1 h mode, however, force recovery diminished widely, as indicated by the observed percentages (Table 2).

### Plastic deformation

**Fig. 7:**
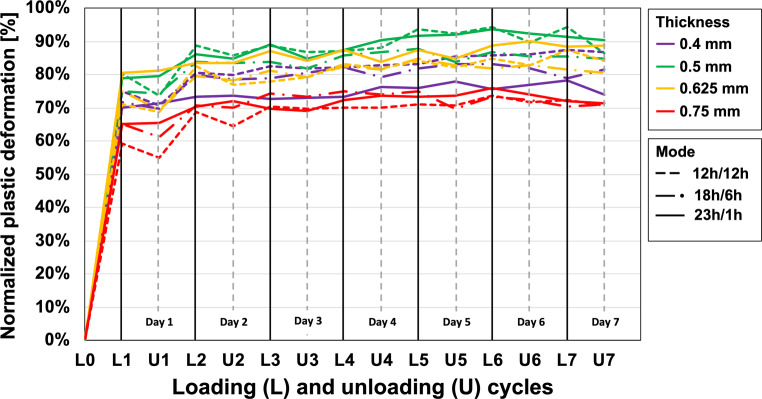
Play induced by plastic deformation of the different specimen thicknesses for the different loading/unloading modes during the 7‑day observation period Durch plastische Verformung induziertes Spiel der verschiedenen Probendicken für die verschiedenen Be‑/Entlastungsarten während des 7‑tägigen Beobachtungszeitraums

As shown by the median curves (Fig. 7), most plastic deformation of the specimens also occurred during the first loading interval (L1). Taking the thickness-dependent maximum deflection ranges as 100% reference, grand median percentages at L1 were 72, 79, 75, and 65% for the 0.4-mm, 0.5-mm, 0.625-mm, and 0.75-mm specimens, respectively (Table 3).

**Table 3:** Individual deflection values and normalized plastic deformation values for the different specimen thicknesses. The values 12 h/12 h, 18 h/6 h, and 23 h/1 h denote the different loading/unloading modes, i.e., 12 h/12 h, 18 h/6 h, and 23 h/1 h, respectively. The values in parentheses correspond to the maximum and minimum values for the corresponding normalized offset in percent Einzelne Durchbiegungswerte und normalisierte plastische Verformungswerte für die verschiedenen Probendicken. Die Werte 12 h/12 h, 18 h/6 h und 23 h/1 h bezeichnen die verschiedenen Be‑/Entlastungsmodi, d. h. 12 h/12 h, 18 h/6 h und 23 h/1 h. Die Werte in Klammern entsprechen den Maximal- und Minimalwerten für den entsprechenden normierten Offset in Prozent

Film thickness		0.4 mm			0.5 mm			0.625 mm			0.75 mm		
*Loading/unloading mode*		12 h/12 h	18 h/6 h	23 h/1 h	12 h/12 h	18 h/6 h	23 h/1 h	12 h/12 h	18 h/6 h	23 h/1 h	12 h/12 h	18 h/6 h	23 h/1 h
*Individual deflection [mm]*		0.2	0.2	0.2	0.15	0.15	0.15	0.15	0.15	0.15	0.1	0.1	0.1
*Normalized plastic deformation [%]*	*L0*	0%	0%	0%	0%	0%	0%	0%	0%	0%	0%	0%	0%
	*L1*	75.0% (71–75)	71.8% (71–75)	70.0% (70–72)	80.3% (73–81)	74.9% (74–78)	78.9% (74–83)	71.1% (70–75)	75.0% (73–82)	80.5% (71–82)	59.2% (59–66)	65.3% (61–65)	65.2% (61–67)
	*U1*	70.7% (70–74)	69.7% (66–74)	71.5% (70–73)	73.8% (69–76)	74.3% (73–75)	79.8% (76–83)	68.9% (68–73)	69.5% (68–79)	81.3% (71–82)	55.2% (54–62)	61.1% (58–62)	65.4% (62–66)
	*L2*	80.5% (79–81)	79.8% (79–83)	73.5% (72–75)	88.6% (83–90)	83.9% (83–85)	86.2% (79–89)	82.4% (77–85)	79.6% (78–86)	83.6% (75–89)	68.8% (68–76)	70.7% (66–73)	70.6% (66–72)
	*U2*	79.9% (79–82)	78.7% (78–79)	73.7% (72–76)	85.9% (81–87)	83.6% (79–85)	85.0% (79–87)	77.1% (75–79)	78.3% (77–85)	83.6% (75–90)	64.7% (64–73)	70.1% (65–72)	72.0% (68–72)
	*L3*	82.7% (82–85)	78.9% (79–79)	72.6% (70–75)	88.8% (82–91)	84.0% (83–85)	89.0% (79–90)	78.1% (78–83)	81.2% (80–87)	87.1% (75–88)	70.3% (69–78)	74.4% (71–75)	69.7% (69–74)
	*U3*	82.1% (81–83)	80.6% (76–81)	73.1% (73–76)	86.8% (84–89)	81.8% (81–86)	85.0% (79–88)	79.2% (78–81)	79.2% (78–86)	84.1% (73–89)	69.7% (68–76)	73.2% (68–76)	69.2% (69–73)
	*L4*	82.2% (82–85)	82.4% (82–82)	73.5% (73–75)	87.1% (83–88)	85.8% (85–87)	87.4% (79–90)	83.3% (80–84)	82.7% (80–89)	87.4% (76–92)	70.0% (69–77)	74.9% (70–79)	72.3% (72–74)
	*U4*	82.9% (82–85)	79.2% (79–82)	76.3% (75–78)	88.2% (84–90)	86.9% (85–87)	90.4% (81–91)	81.8% (80–83)	81.7% (80–87)	84.0% (77–91)	70.1% (69–76)	74.2% (68–78)	73.6% (71–76)
	*L5*	83.2% (82–84)	81.8% (82–83)	76.0% (75–77)	93.8% (88–94)	87.9% (84–88)	91.8% (82–93)	84.3% (81–87)	84.9% (82–90)	87.6% (77–90)	71.2% (71–77)	75.0% (73–79)	73.5% (73–78)
	*U5*	85.7% (84–86)	83.4% (76–83)	77.9% (78–78)	92.3% (88–93)	84.0% (80–85)	92.1% (81–95)	82.1% (81–83)	83.0% (79–86)	84.9% (78–90)	70.9% (69–74)	69.7% (69–76)	73.8% (72–76)
	*L6*	85.7% (85–87)	83.1% (81–86)	75.7% (75–76)	94.3% (89–95)	86.9% (85–87)	93.9% (83–94)	84.8% (80–90)	81.9% (81–89)	88.9% (79–94)	73.7% (71–80)	73.5% (70–78)	75.9% (75–77)
	*U6*	86.1% (86–86)	82.1% (82–82)	76.9% (77–78)	89.8% (88–92)	85.5% (84–87)	92.3% (86–95)	83.0% (81–90)	83.0% (80–89)	90.1% (78–95)	71.9% (69–77)	72.5% (66–74)	74.0% (70–77)
	*L7*	87.5% (87–88)	78.9% (78–80)	78.2% (76–79)	94.2% (91–94)	85.5% (84–86)	91.3% (82–93)	87.6% (83–92)	81.7% (78–85)	88.5% (80–92)	72.4% (72–79)	70.5% (69–76)	72.0% (70–77)
	*U7*	87.0% (85–88)	81.7% (81–82)	74.0% (74–78)	86.5% (86–88)	84.9% (83–86)	90.6% (85–94)	84.0% (80–88)	80.6% (80–82)	88.7% (78–93)	70.7% (69–76)	71.1% (68–76)	71.4% (68–73)

Normalized plastic deformation reached a near plateau after L4, with a median value of 83% for all specimen thicknesses and modes. Comparison of the normalized plastic deformation for the three loading/unloading modes reveals no systematic differences among them.

### Stiffness values

**Fig. 8:**
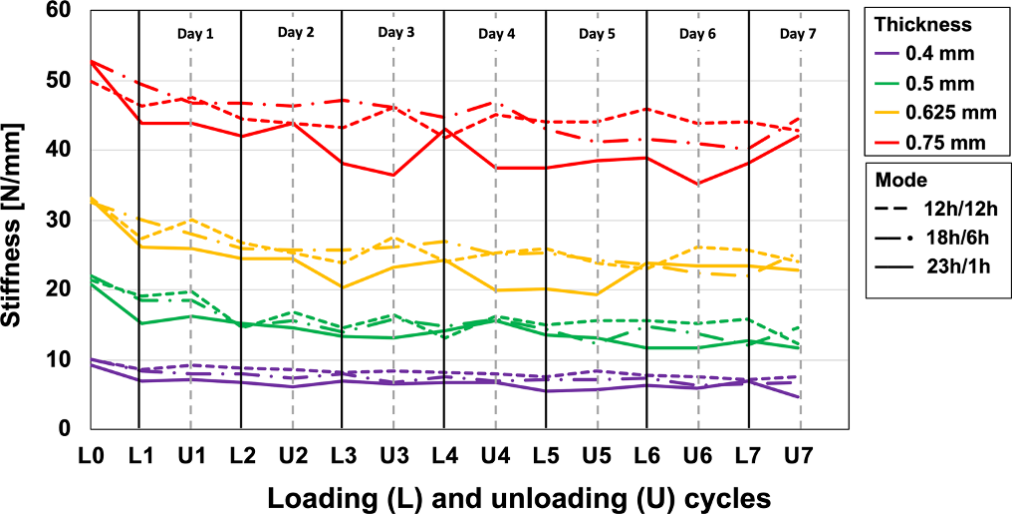
Stiffness values for different PET‑G thicknesses for the different loading/unloading modes during the 7‑day observation period Steifigkeitswerte für verschiedene PET-G-Dicken für die verschiedenen Be- und Entlastungsarten während des 7‑tägigen Beobachtungszeitraums

As revealed by Fig. 8, the stiffness values of a loaded specimen tended to decrease, with median reductions of 16.2, 25.5, and 28.1% recorded at L1, L4, and L7, respectively (median values over all thicknesses and modes). This tendency was, however, somewhat unsystematic, i.e., no clear or statistically significant interdependencies with specimen thickness or loading/unloading mode were observed (Fig. 8 and Table 4).

**Table 4:** Initial and normalized stiffness for the different specimen thicknesses. The values 12 h/12 h, 18 h/6 h, and 23 h/1 h denote the different loading/unloading modes, i.e., 12 h/12 h, 18 h/6 h, and 23 h/1 h, respectively. The values in parentheses correspond to the maximum and minimum values for the corresponding stiffness in N/mm and normalized stiffness in percent Initiale und normierte Steifigkeiten für die verschiedenen Probekörperdicken. Die Werte 12 h/12 h, 18 h/6 h und 23 h/1 h bezeichnen die verschiedenen Be‑/Entlastungsmodi, d. h. 12 h/12 h, 18 h/6 h und 23 h/1 h. Die Werte in Klammern entsprechen den Maximal- und Minimalwerten für die entsprechende Steifigkeit in N/mm und die normierte Steifigkeit in Prozent

Film thickness		0.4 mm			0.5 mm			0.625 mm			0.75 mm		
*Loading/unloading mode*		12 h/12 h	18 h/6 h	23 h/1 h	12 h/12 h	18 h/6 h	23 h/1 h	12 h/12 h	18 h/6 h	23 h/1 h	12 h/12 h	18 h/6 h	23 h/1 h
*Initial stiffness [N/mm]*		10.1 (10.3–9.9)	10.1 (10.3–9.8)	9.3 (9.4–9.0)	21.4 (21.8–20.8)	21.9 (22.7–21.9)	20.9 (21.9–20.8)	33.1 (33.1–32.6)	32.5 (33.8–30.6)	32.9 (33.1–31.8)	49.9 (51.4–48.9)	52.8 (53.3–51.3)	52.6 (53.2–52.6)
*Normalized stiffness [%]*	*L0*	100%	100%	100%	100%	100%	100%	100%	100%	100%	100%	100%	100%
	*L1*	84.1% (83–87)	82.4% (70–85)	75.3% (68–77)	89.6% (86–93)	84.3% (76–87)	72.9% (70–83)	82.8% (82–86)	92.4% (83–93)	79.5% (75–85)	93.0% (86–95)	93.7% (84–96)	83.5% (73–89)
	*U1*	91.5% (90–92)	79.0% (72–84)	77.4% (58–79)	92.2% (92–95)	83.9% (80–85)	78.0% (74–81)	90.8% (90–93)	86.2% (83–86)	78.8% (70–84)	95.3% (93–96)	88.6% (81–93)	83.5% (72–91)
	*L2*	86.2% (69–88)	78.0% (64–82)	73.5% (66–84)	68.3% (65–79)	67.4% (67–70)	73.3% (56–78)	80.9% (69–82)	79.8% (77–85)	74.8% (68–82)	89.2% (85–94)	88.3% (74–93)	79.9% (73–86)
	*U2*	84.8% (80–88)	72.6% (66–79)	64.7% (62–76)	79.1% (76–82)	70.8% (62–72)	69.4% (55–73)	76.4% (75–82)	79.3% (76–82)	74.6% (67–86)	87.7% (85–95)	87.8% (75–96)	83.6% (67–90)
	*L3*	81.3% (72–88)	79.4% (62–85)	74.0% (69–84)	68.1% (68–71)	63.9% (62–64)	64.2% (62–76)	72.4% (64–85)	78.9% (78–82)	61.8% (62–73)	86.4% (78–93)	89.3% (68–95)	72.3% (69–81)
	*U3*	83.1% (80–88)	66.4% (61–85)	69.5% (66–78)	76.4% (71–85)	72.1% (68–74)	63.1% (56–65)	83.3% (77–85)	80.5% (79–82)	70.5% (59–73)	92.3% (82–94)	87.2% (72–96)	69.1% (64–84)
	*L4*	80.8% (70–82)	74.8% (63–80)	72.5% (60–79)	61.0% (46–79)	67.0% (59–73)	68.0% (62–77)	72.4% (72–86)	82.6% (77–88)	74.1% (60–78)	83.9% (79–93)	84.4% (74–92)	81.7% (56–85)
	*U4*	77.8% (72–86)	68.9% (63–78)	73.6% (62–74)	76.3% (70–79)	72.1% (67–75)	74.8% (67–75)	76.7% (73–84)	77.2% (71–82)	61.0% (58–73)	90.1% (80–95)	88.9% (85–92)	71.3% (66–82)
	*L5*	74.4% (69–84)	70.5% (64–80)	59.2% (49–68)	70.4% (66–75)	65.2% (65–66)	65.2% (60–70)	78.6% (72–87)	77.6% (70–83)	61.3% (61–63)	88.4% (73–94)	81.6% (72–98)	71.4% (69–81)
	*U5*	82.3% (81–85)	70.0% (65–83)	62.0% (59–62)	72.7% (68–75)	56.6% (48–63)	62.9% (50–65)	72.2% (64–86)	74.6% (72–76)	58.6% (57–63)	88.1% (73–93)	77.9% (73–88)	73.2% (71–77)
	*L6*	77.5% (68–84)	72.0% (64–79)	68.7% (50–78)	72.8% (68–77)	67.5% (63–68)	56.5% (56–65)	69.5% (69–79)	72.5% (70–77)	72.9% (71–75)	91.8% (75–94)	78.9% (72–90)	73.9% (68–86)
	*U6*	74.9% (69–81)	63.4% (53–73)	62.5% (59–68)	71.4% (70–73)	62.3% (52–67)	56.3% (53–59)	78.8% (78–83)	69.1% (63–82)	71.3% (62–77)	88.0% (77–92)	77.5% (67–81)	67.0% (63–76)
	*L7*	71.1% (64–83)	63.9% (54–67)	74.0% (65–79)	74.4% (53–79)	55.5% (54–61)	61.4% (58–62)	77.9% (76–85)	67.5% (62–73)	71.5% (71–79)	88.2% (80–90)	76.0% (69–89)	72.4% (69–85)
	*U7*	74.8% (64–77)	66.1% (65–74)	51.4% (46–64)	57.2% (46–72)	66.2% (54–66)	55.9% (51–62)	73.0% (68–78)	77.5% (65–79)	69.6% (64–77)	85.9% (80–92)	84.2% (72–90)	80.0% (68–80)

## Discussion

During the typical intraoral application period for an aligner of 7–10 days, aligner materials are exposed to saliva and are constantly loaded due to the discrepancy between the “programmed” tooth position on the setup model and the actual tooth position. Many studies have investigated the effect of loading on aligner materials for short periods of up to 24 h [10, 21, 24]. Only one has studied how different deflection ranges affect the stress relaxation of thermoformed aligners over a longer period, examining 1.0-mm-thick aligners under constant loading for 14 days [20]. The results indicated rapid stress relaxation within the first 8 h, followed by a lower rate of reduction until a plateau-like level was reached after the fourth or fifth day. These studies provided important insights into time-dependent force delivery under constant material loading. However, they did not address the fact that aligners are not worn constantly, but are usually inserted and removed several times throughout the day. This loading and unloading schedule is, to a certain extent, repeated daily and exhibits inter-individual variability depending on the patient’s lifestyle and treatment compliance. By examining different loading/unloading modes, we aimed to simulate different clinically representative scenarios in order to clarify their effect on the forces exerted by aligners on individual teeth. The PET‑G specimens tested in our study clearly showed the largest force decay (i.e., stress relaxation) during the first loading period, followed by smaller reductions until a near-constant residual force value was reached. This is in accordance with previous studies in which constant loading of thermoplastic film specimens also yielded a very high rate of force reduction during the first 8 h of constant loading [20, 21]. The inclusion of loading and unloading periods in the study protocol revealed that, irrespective of the specific loading/unloading mode, PET‑G specimens exhibit a certain force-recovery pattern after unloaded periods, which is most prominent in the first loading cycle and then decreases progressively during subsequent cycles (Fig. 6b). Such long-term and load-dependent force–deflection behavior is mainly due to the viscoelastic behavior of PET‑G aligner materials. More specifically, such materials exhibit both elastic and time-dependent viscous properties when subjected to load. This behavior has been mathematically explained by means of the standard linear solid model, which consists of springs and pistons representing both the elastic and viscous properties, respectively [23]. The viscous characteristics are manifested in the time-dependent plastic deformation of the tested specimens (Fig. 7; Table 3). The specific plastic deformation values are clearly not only time-dependent, but also depend on the effective deflection range (d_eff_), which can be calculated by subtracting play due to plastic deformation (d_pd_) from the predefined thickness-dependent deflection (d_thick_; Eq. 1).

1 $$\mathrm{d}_{\mathrm{eff}}=\mathrm{d}_{\text{thick}}-\mathrm{d}_{\mathrm{pd}}$$

The reduction of effective deflection after loading described by Eq. 1 is important for the interpretation of our results. For example, the 0.5-mm specimens were initially deflected by 0.15 mm, creating a local stress of approximately 14.41 MPa [8]. After the first loading/unloading cycle, plastic deformation was 0.13 mm. This means that during the second loading/unloading cycle, the 0.5-mm specimens were effectively deflected by only 0.02 mm, resulting in stress values of only 1.92 MPa. Such stress reduction from cycle to cycle explains the plateau-like behavior of both the deflection force and plastic deformation. Our measurements indicate that the reduction in PET‑G material stiffness was much smaller, as illustrated by the flatness of the lines in Fig. 8. The somewhat unsystematic fluctuations in stiffness after a clear initial reduction may be explained by the subsequent, small effective deflections (d_eff_) ranging between 0.01 and 0.06 mm, which also resulted in fewer points to apply a linear fit to the terminal curve portions.

In vitro studies performed with similar aligner materials thermoformed on dentition models indicated very large initial forces for different types of tooth movement, with values exceeding the forces required for the types of movement investigated [4–7, 12, 14, 16, 17]. With regard to the actual load magnitudes applied during clinical therapy, however, two main factors must be considered, both of which reduce the forces and moments applied to individual teeth: (i) the elasticity of the PDL, which initially reduces the effective discrepancy between the tooth’s position on the setup model and that in the aligner, and (ii) the stress relaxation phenomenon observed in the current study. The latter can also be expected, although with a different extent, for other aligner materials due to the viscoelasticity of the polymers used [21]. In mathematical terms, forces applied by aligner materials to individual teeth can be deduced from the effective discrepancy *x(t)* between the actual tooth position and the programmed position (*z*_setup_) after subtracting the time-dependent initial tooth movement *(tm*_*i*_*)* and the time-dependent plastic deformation of the aligner *(d*_*pd*_*)*. This discrepancy can be calculated as follows:

2 $$x\left(t\right)=z_{\text{setup}}-d_{pd}-tm_{i}$$

In turn, the effective forces *F(t)* can be calculated as follows:

3 $$F\left(t\right)=x\left(t\right)\times k\left(t\right)$$

where *x(t)* is the effective discrepancy and *k(t)* is the stiffness of the aligner.

From a clinical point of view, these factors might explain why the amount of root resorption observed after treatment with PET‑G aligners is slightly larger than that for light orthodontic forces [1]. However, it should be noted that aligners fabricated from less stiff or multi-layered aligner materials might induce less external root resorption when compared to orthodontic treatment with fixed appliances [11].

The residual forces at the end of the observation period differed among the three loading/unloading modes. Specifically, residual forces for the 12 h/12 h loading/unloading mode were significantly larger than those for the 23 h/1 h loading mode (Mann–Whitney *U* test, *p* < 0.05; Table 2). This difference is probably related to (i) longer loading times and, in turn, more plastic deformation of the aligner material, and (ii) shorter unloading periods, reducing the length of time for material recovery. Therefore, our initial hypothesis was accepted, indicating that longer loading cycles will result in greater stress relaxation and larger initial force reductions than shorter loading cycles. Although equal stresses were applied to the different specimen thicknesses, the effect was thickness-dependent, with the largest normalized residual forces recorded for the 0.75-mm specimens (Mann–Whitney *U* test, *p* < 0.01).

This in vitro study considered the effect of body temperature and water immersion on the force delivery of PET‑G specimens. Further studies, however, should be performed on the newly developed multilayered aligner materials (e.g., SmartTrack, Align Technology, Santa Clara, CA, USA; Zendura™ FLX, Bay Materials LLC, Fermont USA or CA-Pro, Scheu Dental GmbH, Iserlohn, Germany), due to their potentially different behavior in the intraoral environment and different stress relaxation patterns [2, 3, 26].

It is important to note that flat probes do not replicate the geometry of a real aligner. However, in vitro measurements with flat specimens not only enable simpler mechanical testing but also easier interpretation of the test results; this is because they limit the number of influencing factors, e.g., the complex geometry of aligners thermoformed on dentition models. A general limitation of investigating flat material specimens is that the measured force magnitudes cannot be directly compared with those found in clinical application due to the complex effect of the geometry of the three-dimensional dental arch. Therefore, we included only the normalized force changes and normalized plastic deformation for the further analysis of the measured data instead of the raw force values. Nonetheless, further studies are required to investigate how such geometry affects force decay, stiffness change, and plastic deformation. Moreover, according to our power analysis the selected sample size (*n* = 3) was sufficient to statistically evaluate the differences of the force reduction after the first loading cycle for the different thicknesses and loading/unloading modes. Along the further loading cycles, a higher sample size (*n* = 17) would have been required to evaluate the difference between the different loading/unloading modes. However, due to the variability and overlapping of the force reduction values we considered these differences as clinically irrelevant, omitting the need to include further samples.

## Conclusions

During long-term loading of aligner materials, plastic deformation of up to 83% should be expected without affecting material stiffness. The highest force reduction could already be observed after the first loading interval. Moreover, we found that the longest investigated simulated intraoral application period of 23 h per day induced the largest decay in force, with residual forces after one week of a mere 5% of those initially applied. The observed force reduction, in addition to the constantly decreasing discrepancy between the programmed tooth position in the aligner and the actual tooth position, will attenuate the initially applied excessive forces observed in previous in vitro studies.
